# Facing the fear: a narrative review on the potential of pressure training in music

**DOI:** 10.3389/fpsyg.2024.1501014

**Published:** 2024-12-13

**Authors:** Nicky de Bie, Yannick Hill, J. R. (Rob) Pijpers, Raôul R. D. Oudejans

**Affiliations:** ^1^Department of Human Movement Sciences, Vrije Universiteit Amsterdam, Amsterdam, Netherlands; ^2^Amsterdam Movement Sciences, Amsterdam, Netherlands; ^3^Institute of Brain and Behaviour, Amsterdam, Netherlands; ^4^Lyda Hill Institute for Human Resilience, University of Colorado Colorado Springs, Colorado Springs, CO, United States; ^5^Center of Expertise Urban Vitality, Amsterdam University of Applied Sciences, Amsterdam, Netherlands

**Keywords:** anxiety, exposure, learning, performance, stress, resilience

## Abstract

Music performance anxiety (MPA) is one of the most reported psychological problems among musicians, posing a significant threat to the optimal performance, health, and psychological wellbeing of musicians. Most research on MPA treatment has focused on reducing symptoms of performance anxiety, but complete “cures” are uncommon. A promising addition or alternative that may help musicians enhance their performance under pressure, despite their anxiety, is pressure training (PT). In other high-pressure domains, such as sports and police work, pressure training has been proven effective in reducing choking and enhancing performance quality under pressure. Therefore, the aim of this narrative review is to explore the potential of pressure training in music settings. Specifically, we first provide a theoretical overview of current models explaining performance declines due to anxiety. Second, we discuss the current state of research on the effectiveness and application of pressure training in sports and police work as well as recent developments in pressure training interventions for music settings. While there is a limited number of studies investigating the effectiveness of pressure training on musicians' performance quality, research focusing on musicians' experiences has shown that pressure training can be particularly beneficial for enhancing performance skills, preparing for performances, and managing performance anxiety. Based on the reviewed literature, the final section points out suggestions for future research as well as recommendations for musicians, teachers, and music institutions for practical applications.

## 1 Introduction

Feeling sweaty palms, a trembling voice, and an accelerated heartbeat is a common experience for musicians playing in front of an audience or during an audition. Performing under high-pressure conditions, where the consequences of mistakes are evident, can induce anxiety, often leading to a decline in performance (Papageorgi et al., 2013; Steptoe and Fidler, 1987; Wan and Huon, 2005). Research on professional musicians and music students shows that music performance anxiety (MPA) is one of the most reported psychological problems (Williamon and Thompson, 2006), with studies indicating prevalence rates ranging from 16.5 to 60% (Fernholz et al., 2019). For example, Van Kemenade et al. (1995) found that among members of professional orchestras 55% had experienced performance anxiety seriously enough that it affected their professional lives considerably. Hildebrandt et al. (2012) performed a longitudinal study including 105 music students and showed a significant increase in fatigue, depression and stage fright within the 1st year after attending the music university. Furthermore, Orejudo Hernández et al. (2018) showed that 33.9% of music students used substances to cope with MPA, and 19% considered dropping out because of these feelings of anxiety. This persistent and overwhelming anxiety experienced during musical performances in both educational and professional settings poses a significant threat to the optimal performance, health, and psychological wellbeing of musicians (Kenny, 2011; Kenny et al., 2014).

MPA can be defined as follows:

Music performance anxiety is the experience of marked and persistent anxious apprehension related to musical performance that has arisen through specific anxiety conditioning experiences and which is manifested through combinations of affective, cognitive, somatic and behavioral symptoms. It may occur in a range of performance settings, but is usually more severe in settings involving high ego investment and evaluative threat. It may be focal (i.e., focused only on music performance), or occur comorbidly with other anxiety disorders, in particular social phobia. It affects musicians across the lifespan and is at least partially independent of years of training, practice and level of musical accomplishment. It may or may not impair the quality of the musical performance (Kenny, 2009, p. 433).

The debilitating effects of MPA can manifest in a combination of various symptoms, including negative affect (e.g., feelings of uncontrollability, hopelessness or depression; Kenny, 2011), irrational or catastrophic cognitions about making mistakes or audience evaluation (Kenny and Osborne, 2006; Osborne et al., 2014), autonomic arousal such as trembling and hyperventilation, and behavioral responses like the avoidance of performances and auditions, overt expressions of anxiety, and a serious decline in performance quality (Salmon, 1990). This occurrence, where anxiety diminishes performance quality below one's expected capabilities, is often referred to as choking under pressure (Baumeister, 1984).

Over the past three decades, most research on MPA treatment has focused on reducing these various perceived symptoms of performance anxiety, trying to provide musicians with strategies to cope with their fears. For example, cognitive behavioral therapy, focusing on modifying faulty cognitions and changing problematic behaviors (Beck, 2011), has been shown to be effective in treating MPA, and is widely considered to be the treatment of choice (Kenny, 2005). Moreover, the use of betablockers is considered to be particularly suited for treating physiological symptoms of MPA, such as sweating palms, palpitations, and tremors (Brandfonbrener, 1990). However, such medications seem to come with numerous adverse side effects and there is no clear evidence that they enhance the quality of performance (Kenny, 2011). Furthermore, there has been growing interest in mental skills training (Cohen and Bodner, 2019), acceptance and commitment therapy (Clarke et al., 2020), and the use of exercise (Stern et al., 2012), to reduce anxiety and increase coping skills in musicians.

Empirical evidence indicates that the combination of cognitive and behavioral approaches yields the best outcomes for reducing MPA, although complete “cures” are uncommon (Kenny, 2006). Most forms of MPA have proven to be challenging to treat, and post-treatment anxiety levels rarely decrease to those of non-anxious individuals (for a review, see Kenny, 2005, 2006). All these forms of treatment have in common that they focus on symptom reduction by trying to alleviate the fear, which is rarely deemed entirely successful, which is confirmed by the recent review of Herman and Clark (2023). They showed that the prevalence rates of MPA have remained unchanged since the first large-scale studies in the 1980's, indicating that current approaches overall do not have a meaningful impact (Herman and Clark, 2023). Furthermore, only a small number of musicians actually seeks professional help when experiencing MPA (Burin et al., 2019). Musicians often face challenges in discussing the psychological aspects of music performance, largely due to a lack of emphasis on mental health among their peers and the prevailing belief that MPA is a normal and natural part of their profession (Kenny, 2011; Perkins et al., 2017). This persistent stigma surrounding MPA makes it difficult for musicians to share their problems and seek specialized treatment (Burin et al., 2019; Cohen and Bodner, 2019). Therefore, instead of defining MPA as a pathological condition, Herman and Clark (2023) propose reconceptualizing it as a normal and natural response to the stressors of performance and the uncertainties of a competitive profession. This shift in perspective could reshape how MPA is understood and encourage new approaches to supporting musicians throughout their careers. Consequently, it could be beneficial, in addition to the existing treatments, to increase our understanding of methods that help musicians enhance their performance under pressure, despite their anxiety (Fletcher and Arnold, 2021; Kegelaers et al., 2022).

One type of intervention that has been proven to be effective to counter the negative effects of choking by increasing performance quality under pressure, is pressure training (PT; Fletcher and Arnold, 2021; Kegelaers and Oudejans, 2024; Low et al., 2021). This intervention involves developing and practicing skills under high-pressure conditions, aiming to enable individuals to achieve and maintain peak performance under pressurized circumstances (Fletcher and Arnold, 2021). Within other achievement contexts, such as sports and police work, there is initial evidence for the effectiveness of PT. Findings suggest that PT prevents choking under pressure, and consequently has a positive influence on enhancing performance under pressure (for a meta-analysis, see Low et al., 2021). Reducing negative outcomes from stress and pressure could also alleviate some of the mental health issues mentioned by Kenny (2011), and Kenny et al. (2014) in the long-run.

However, less research has been done into PT for musicians suffering from MPA. Questions remain as to how findings from other high-pressure domains, such as sports and police work, generalize to a music setting and what effective methods can be used to implement pressure in this context. Therefore, the aim of this narrative review is to explore the potential of pressure training in music settings. Given the limited number of studies on PT in music, a narrative review was chosen to provide a broad exploratory overview of PT's development. This approach involved systematically searching for studies on PT across various high-pressure domains (see next section), synthesizing the findings, and transferring this knowledge to explore its potential applications in the field of music (Grant and Booth, 2009). After the search strategy, we first provide a theoretical overview of the underlying mechanisms of choking under pressure. Subsequently, this review will give an overview of studies examining PT in sports and police work, followed by a discussion of practical ways of implementing PT in a sports context. Then, the current state of research regarding the effectiveness and application of PT in music settings will be presented. Finally, suggestions for future research as well as practical applications for musicians, music teachers and music institutions will be discussed.

## 2 Search strategy

Our search strategy was split into four steps (see Appendix for more elaborate details). In the first step, we searched specifically for MPA with regards to its definition, prevalence, or treatment methods. In the second step, we expanded our search to choking under pressure in sports and police work to obtain an overview of theoretical mechanisms that could be relevant for MPA. Given that much of this work is associated with fine motor skills, we explicitly included search terms of popular theoretical approaches in this domain (e.g., Integrated Model of Anxiety and Perceptual-Motor Performance, Nieuwenhuys and Oudejans, 2012, 2017). In the third step, we searched for pressure training interventions that have been applied in both the sports and police domain. Finally, we explored whether elements of pressure training have been applied in music. Note that for all steps, specific authors who have a track record of publishing in these various domains were also included. Furthermore, references that were provided in identified reviews and meta-analyses were used to further expand the search.

## 3 Theoretical overview of choking under pressure

When performing under pressure, it is not uncommon that performance deteriorates due to the experienced anxiety, often referred to as choking under pressure (Baumeister, 1984). Explanations for this decline in performance can be found in the attentional theories of choking, such as self-focus and distraction theories. The self-focus theories (e.g., explicit monitoring, Beilock and Carr, 2001; reinvestment, Masters, 1992) propose that anxiety heightens self-consciousness which may cause attention to turn inward, leading to explicitly monitoring and step-by-step control of skill execution. In expert performers, this increased focus on their movements disrupts automatic skill execution, with choking as a result. Alternatively, and increasingly proposed and investigated in the last decade, the distraction theories, like the Attentional Control Theory (Eysenck et al., 2007) and the Integrated Model of Anxiety and Perceptual-Motor Performance (Nieuwenhuys and Oudejans, 2012, 2017) argue that anxiety diverts more attentional resources toward detecting threat-related stimuli in anxiety-inducing situations, at the expense of focusing attention on task-related stimuli. That is, task-related attention is distracted and drawn away by task-irrelevant information.

This impairment of attentional control can be explained by a disruption in the balance between two attentional systems, namely the goal-directed and the stimulus–driven attentional system. The goal-directed system is guided by current goals and expectations, whereas the stimulus-driven attentional system reacts to salient or unexpected stimuli (Corbetta and Shulman, 2002). Anxiety caused by the pressure to perform well leads to an increase in the stimulus-driven attention system and a decrease in the goal-directed attention system. In other words, a shift in attention toward threat-related but task-irrelevant stimuli takes place making it more difficult to focus on task-relevant information (Eysenck et al., 2007; Nieuwenhuys and Oudejans, 2012, 2017). The task-irrelevant stimuli that draw attention away from the task can be both internal and external. For example, in case of musicians, one can be distracted by internal worries and disturbing thoughts about the negative consequences of failing, but also by external cues, such as reactions from an audience or an audition committee (Buma et al., 2015; Osborne et al., 2014).

Over the years, studies in several high-pressure domains, such as sports and police work, confirmed that anxiety-inducing pressure can result in an increased focus on threat-related stimuli leading to a drop in performance. For example, Nieuwenhuys and Oudejans (2010) conducted an experiment investigating the underlying mechanisms of performance deterioration during a stressful shooting task by police officers. The study included seven police officers and two distinct experimental conditions: a low-anxiety scenario, facing a non-threatening opponent and a high-anxiety scenario, encountering a threatening opponent who had the possibility of firing back with colored soap cartridges. The findings demonstrated a statistically significant decline in shooting accuracy under high-anxiety conditions. This decline was attributed to heightened reaction speed at the expense of accuracy, altered head and body positioning (such as making oneself smaller and turning away from the opponent while reloading), and an increased blink rate. These results suggest that under high-anxiety conditions, attention may have shifted away from the task toward the perceived sources of threat (Nieuwenhuys and Oudejans, 2010). This is confirmed by Wilson et al. (2009) who showed that, when anxious, soccer players taking penalties have an increase in the speed at which their gaze was drawn toward the goalkeeper, reflecting an attentional bias toward this threat related target. They also fixated their gaze longer on the goalkeeper, resulting in a noticeable decline in shooting accuracy due to shots that were significantly more centralized and within the goalkeeper's reach.

Following these studies that investigated attention by observing behavioral characteristics in high and low anxiety conditions, Oudejans et al. (2011) explored the thoughts and focus of attention of elite athletes in a variety of sports when performing under pressure. They asked 70 athletes, with on average 5.6 years of experience at the highest level of competition, to complete questionnaires about their thoughts and attention during peak moments. The key findings revealed a high percentage of statements related to worries (25.9%) in combination with a surprisingly low percentage of statements regarding movement execution (4.1%). These findings align with distraction theories of choking, which state that anxiety shifts attention to worries, drawing attention away from task execution.

More recently, Buma et al. (2015) replicated this study among elite musicians, aiming to explore the attentional processes of musicians in high pressure situations. They found that experienced musicians focus on music-related information, physical aspects and thoughts that give confidence in order to maintain a high level of performance under pressure. However, compared to the athletes (Oudejans et al., 2011), these elite musicians seemed to be less distracted, and they managed quite well to stay focused on task-relevant cues even while performing under pressure. The authors suggested that these findings could be explained by the elite musicians' extensive professional experience [on average, 24 years compared to the 5.6 years of expert experience of the athletes in Oudejans et al. (2011)].

In a follow-up study, Oudejans et al. (2017) investigated a less experienced population of music students. They performed two studies in which they explored music students' thoughts and attention on three different moments in time. First, when performing under pressure, second, just prior to making a mistake, and finally, after making a mistake once choking had occurred. The results showed that more task-irrelevant thoughts (i.e., worry and disturbing thoughts) were present just prior to choking, which also meant that the students had less attention for music-related information at that particular moment. These outcomes are in line with the earlier described distraction theories on anxiety and performance (Eysenck et al., 2007; Nieuwenhuys and Oudejans, 2012, 2017), proposing that anxiety evokes a shift in attention from task-relevant information toward task-irrelevant information.

In summary, it can be concluded that while performing under pressure, attention may shift from the primary task to irrelevant stimuli. This phenomenon occurs across various high-performance domains, including athletes, police officers, and musicians. Considering the fact that this attention shift occurred much less frequently in experienced musicians than in music students suggests that extensive experience in performing under pressure trains musicians to maintain better focus on their task. This aligns with findings from Papageorgi et al. (2013) and Steptoe and Fidler (1987) who showed that experience has a positive influence on the perceived impact of anxiety on performance. Moreover, these findings would advocate for an intervention in which musicians can regularly practice under pressure to learn how to perform well, despite feelings of anxiety.

## 4 Pressure training in high-pressure domains

A shift in attention from task-relevant to task-irrelevant information not only occurs when musicians have to perform under pressure, but also in other high-pressure domains, such as sports and police work. It has repeatedly been shown that anxiety-inducing pressure can result in an increase of focus on threat-related stimuli (see Nieuwenhuys and Oudejans, 2012, 2017). In these high-pressure domains, growing attention has been given to PT, an intervention designed to help individuals perform effectively under anxiety inducing circumstances. In contrast with other interventions that primarily focus on managing or reducing anxiety, PT aims to enhance the ability to perform under pressure by intentionally exposing individuals to simulated stressors in a practice environment (Fletcher and Arnold, 2021). The principles of PT align with more traditional behavioral interventions, such as systematic desensitization and exposure (Bell et al., 2013). These methods have been long used to treat phobias and anxiety disorders by gradually exposing individuals to anxiety-producing thoughts or objects, often starting with imaginary exposure followed by exposure *in vivo* (Wolpe, 1961). Within the context of human performance, Hill et al. (2024) have used the term “behavioral vaccination” to describe the systematic but controlled exposure of individuals to moderate doses of stress to help them withstand larger doses in the future. Controlled exposure to optimize doses is common in training sciences relating to stimulate both physical performance (i.e., supercompensation, Brink et al., 2010) as well as motor learning (Hodges and Lohse, 2022).

### 4.1 Pressure training in police work

Over the past decade, the use of PT as an intervention to enhance performance under pressure has extensively been investigated in various high-pressure domains. To illustrate, Oudejans (2008) aimed to investigate whether practicing in realistic high-pressure scenarios may help prevent a decline in handgun shooting performance for police officers under pressure. In this study, 17 police officers were included and divided in an experimental and a control group. Each participant completed a pre-test under both high- and low-pressure conditions, followed by three 1-h practice sessions, and concluded with a post-test, again under both high- and low-pressure conditions. Shooting took place using colored-soap training cartridges that provide a pain stimulus when hit. During the practice sessions, the experimental group practiced handgun shooting under high pressure by facing an opponent who fired back (with colored-soap training cartridges), and the control group practiced handgun shooting on standard cardboard targets instead of engaging with real opponents. The results showed that, although both groups initially performed worse in front of an opponent occasionally firing back (high pressure) compared to cardboard targets (low pressure), the experimental group's shooting performance no longer deteriorated in the presence of an opponent after the training sessions, whereas the control group's performance remained as impaired as during the pre-test. These findings indicated that reality-based practice under pressure helped police officers maintain their handgun shooting performance in high-pressure situations.

In a follow-up study, Nieuwenhuys and Oudejans (2011) replicated these findings and added a retention test to investigate the long-term effects of PT on shooting accuracy of police officers. They found that the experimental group continued to show positive results at the retention test, which was conducted 4 months after initial training. This suggests that training under anxiety may have not only positive short-term effects, but also beneficial long-term effects on police officers' shooting accuracy under pressure. In addition, they supported their findings with gaze behavior measurements, which revealed that when under anxiety, participants indeed showed an increase in fixations on threat-related sources. However, after practicing under pressure, the experimental group demonstrated more calmness in their gaze and relatively long final fixations on their targets, which was associated with improved performance. The authors argued that training with anxiety led to enhanced self-regulatory processes, enabling individuals to become more effective in directing their attention to task-relevant sources of information.

### 4.2 Pressure training in sports

In the domain of sports, more studies evaluated the effectiveness of PT on performance (for a meta-analysis, see Low et al., 2021). For example, Oudejans and Pijpers (2009) executed two experiments, in which they examined whether training under pressure helps to prevent choking in experienced athletes performing perceptual-motor tasks. In both the first experiment, involving 17 expert basketball players, and the second experiment, involving 17 expert dart players, it was shown that only after training in the high-anxiety condition performance did not deteriorate during a high-anxiety post-test. Alder et al. (2016) investigated international-level badminton players and found that the players assigned to the high-anxiety training maintained accuracy of their anticipation judgements during the high-anxiety post-test, whereas the low-anxiety group showed less accuracy. In addition, PT has been shown to be effective not only among expert athletes, but studies investigating novices also showed these positive effects of practicing with anxiety on performing under pressure (Lawrence et al., 2014; Oudejans and Pijpers, 2010).

According to the Integrated Model of Anxiety and Perceptual-Motor Performance (Nieuwenhuys and Oudejans, 2012, 2017), the positive effect of PT on performance can be attributed to performers' motivation to increase mental effort, which aims to maintain or improve their level of performance when exposed to high levels of pressure and anxiety. In this context, it is expected that individuals will allocate more resources toward inhibiting stimulus-driven impulses and enhancing goal-directed control, striving to restore the balance between the goal-directed and the stimulus–driven attentional system. Thus, PT can counter the negative effects of anxiety on performance as performers learn to direct these additional mental resources toward their task, rather than being preoccupied with the pressure itself (Low et al., 2021). Oudejans and Pijpers (2009) found in their experiments with basketball and dart players that both the control and the experimental groups reported an increase in their mental efforts during the high-anxiety post-test. However, it was observed that only the experimental group's increased efforts led to improved performance after training under pressure, while interestingly, both groups maintained similar levels of anxiety during the post-tests. Therefore, instead of reducing anxiety, PT seemed to help participants acclimatize to perform effectively under anxious conditions, even without the use of additional coping strategies (Nieuwenhuys and Oudejans, 2011).

In conclusion, PT has been shown to be effective in preventing choking and increasing performance under pressure across multiple high-pressure domains, such as sports and law enforcement, and for both experienced and inexperienced performers. Theory and research suggest that through training under pressure increased mental effort can be effectively utilized to maintain a proper attentional balance to achieve optimal performance. Given the positive influence of PT on performance, there is growing attention in research and practice on how to properly implement PT in a safe and ethically sound way (as PT per definition involves taking performers out of their comfort zone during training). That is, the specific dynamics between a given variable that is manipulated to induce pressure and the desired response need to be established precisely to guarantee a safe and ethical training environment (see Hill et al., 2020, 2023, 2024). Thus, PT must not be used as an excuse to arbitrarily expose musicians to any stressor (see Kegelaers et al., 2020; Kegelaers and Oudejans, 2024).

## 5 Applications of pressure training in sports

Considering that PT has proven to be effective in preventing choking under pressure, it is important to increase the understanding of practical and ethical constraints for implementing PT. A key advantage of PT is that it is easily integrated into a performers existing training schedule, because a coach or instructor can increase pressure during an already scheduled exercise (Ellis and Ward, 2022; Low et al., 2021). Research on the effectiveness of PT used various pressure manipulations, proven to be effective in evoking anxiety. Methods directly focusing on the task at hand included monetary rewards (Oudejans and Pijpers, 2009), social judgments (Alder et al., 2016), and forfeits (Bell et al., 2013; Nieuwenhuys and Oudejans, 2011). Other methods directed attention toward increasing athletes' ability to cope with unexpected stressors outside the usual practice environment, such as long travels and poorly organized training camps (Kegelaers et al., 2020). However, it was emphasized that a more comprehensive understanding of the process of implementing PT was essential to guide coaches and performers in effectively and sensitively applying these principles (Kent et al., 2018).

To address this gap, Stoker et al. (2016) explored how pressure is systematically created across sports environments by interviewing 11 professional coaches about their practice. They demonstrated that coaches used two approaches to increase pressure during training, including manipulating the demands of training and the consequences of training. More specifically, coaches manipulated training demands by altering task stressors (e.g., changing guidelines, conditions, and equipment), performer stressors (e.g., inducing fatigue and manipulate tactical information), and environmental stressors (e.g., sounds, temperatures, and visual information). Additionally, consequences can be attached to the task performance through the use of forfeits (cleaning changing rooms; Bell et al., 2013), rewards (e.g., monetary incentives; Oudejans and Pijpers, 2009), and judgment stressors (e.g., evaluations by coaches; Alder et al., 2016). Furthermore, they highlighted the importance of considering individual differences, since coaches believed individual differences to be important in how athletes responded to the created pressure.

Building on this research, Kegelaers et al. (2020) conducted a study in which they interviewed nine high-performance coaches about their techniques for increasing pressure during training using what they termed “planned disruptions.” They found that coaches use a combination of nine distinct themes of planned disruptions to increase pressure and demands during training and preparation, including location (e.g., long travel or poor facilities), competition simulation (e.g., making every exercise a competition), punishment and rewards (e.g., cleaning up the gym or earning more playing time), physical strain (e.g., fatigue), stronger competition (e.g., seeking out stronger opponents), distractions (e.g., noise), unfairness (e.g., referees making bad calls), restrictions (e.g., time, communication or physical restrictions), and outside the box elements (e.g., practicing other sports). By employing these strategies, coaches aimed not only to familiarize athletes with high-pressure situations, but also to cultivate an awareness of their own behaviors under pressure, strengthen mental techniques to handle stress, and enhance team connectivity (Kegelaers et al., 2020).

Although PT has been proven effective on its own (e.g., Nieuwenhuys and Oudejans, 2011), more recent research suggests that combining PT with other interventions and adding moments of reflection after using PT can even further enhance its positive effects, by for example increasing athletes' self-awareness and mastering coping skills (Fletcher and Arnold, 2021; Kegelaers and Oudejans, 2024). To illustrate, Bell et al. (2013) demonstrated that a PT intervention with regular review sessions—discussing what worked well, what needed improvement, and the methods to achieve those improvements— resulted in significant improvements in mental toughness among elite young cricket players, in comparison with the control group.

Other studies combined PT, reflection, and other sport psychological interventions, such as cognitive restructuring and mental skills training (Kent et al., 2022; Low et al., 2023; Van Rens et al., 2021). For instance, Kent et al. (2022) randomly assigned 82 soccer players to either an intervention group (receiving PT, three cognitive behavior workshops, and reflective diaries) or a comparison group (receiving only PT). Results indicated that the players in the intervention group scored significantly higher on decision-making and skill-execution, suggesting additional value of cognitive behavioral workshops and reflective diaries. In addition, Kegelaers et al. (2021) complemented a PT intervention with an introductory resilience development workshop and moments of guided reflection after each training session. Results showed increased awareness in both athletes and coaches, with both groups reporting a desire for more workshops and more moments of explicit reflection, as it facilitated their learning processes.

Van Rens et al. (2021) implemented a 4-week PT program in a state team of 12 female cricket players to optimize athletes' stress responses. Prior to the intervention, two workshops were delivered by the sport psychologist to introduce concepts such as cognitive reappraisal, emotion regulation, and stress exposure. During the program, weekly review moments were organized with coaches, players, and sports psychologists to promote learning. These sessions offered players the possibility to evaluate their response to the stressors, their emotional reaction, and implemented coping strategies. Additionally, they had the opportunity to practice newly learned psychological skills, such as self-talk and attentional control, under pressure. Results showed that the players experienced an increase in awareness of their emotional reactions when confronted with a stressor. The players highlighted the added value of PT, noting that they previously could not practice these skills during regular training sessions, as those sessions did not accurately represent game situations. These findings are in line with Low et al. (2021), who argue that PT can be viewed as a valuable addition to other psychological interventions in sports, as it allows athletes to practice their newly acquired psychological skills in an ecologically valid setting.

Integrating the existing knowledge about implementation of PT, Fletcher and Arnold (2021) were the first to propose a practical guideline for using PT, recommending a multi-phased approach to PT. This approach consists of three phases including preparation and design, delivery and implementation, and debrief and review. During the preparation and design phase, coaches and psychologist must work together to establish aims and desirable outcomes using effective goal-setting strategies, educate themselves about stress in a performance setting, and educate significant others about PT to create a challenging but supportive learning culture (Fletcher and Sarkar, 2016). Furthermore, this phase involves designing the PT by carefully considering the when, where, who, what, and how of its implementation, tailoring the PT design to suit both the specific context and the unique needs of each athlete. Secondly, the delivery and implementation phase should include rehearsing coping strategies and psychological skills, such as imagery and relaxation techniques, in non-pressurized or simulated-pressurized situations, before applying them to actual pressurized training and competition. This approach allows athletes to detect threatening cues earlier and develop appropriate responses (Bell et al., 2013). During practice, the pressure can be gradually increased by using the planned disruptions framework (Kegelaers et al., 2020), constantly monitoring whether desired psychological and performance effects are met. Note, however, that this does not imply that PT should always begin with very low levels of pressure as too small doses are likely to have no effect (see Hill et al., 2024). Especially in domains where specific situations are inevitable, like performing in front of a large crowd for musicians or a soccer player taking a penalty kick during an important match, immediate exposure to these circumstances or a simulation thereof may also be considered. Also, Fletcher and Arnold (2021) emphasize the crucial balance between challenge and support during training, which can be achieved by assessing the delivery of the training and individuals' responses to it. Finally, the third phase of debrief and review, must contain a comprehensive reflection on the entire training process to maximize benefits and learning. The goal of this debrief is to increase insight into how the intervention was delivered and perceived, and to facilitate reflection to maximize the opportunity to learn (Fletcher and Arnold, 2021).

In summary, while PT on its own has proven to be effective in enhancing performance under pressure, recent developments show that it may offer even more benefits, such as mastering coping skills and increasing self-awareness, when embedded in thorough preparation and evaluation.

## 6 Pressure training in music settings

While the influence of PT on choking in sports and police work has already been investigated, fewer studies have been performed in the context of music. Current research in the field of music is increasingly discovering and appreciating the similarities between music and sports contexts. For example, in recent years, sport psychology interventions such as mental training, imagery, and self-talk have been studied for helping musicians to cope with pressure with promising results (Clark and Williamon, 2011; Hoffman and Hanrahan, 2012; Lubert and Gröpel, 2022; Osborne et al., 2014; Spahn et al., 2016). Although these techniques are a valuable and interesting addition to the existing interventions for treating MPA, these methods still primarily focus on alleviating or coping with symptoms of anxiety. Since reducing anxiety is proven to be not always possible or beneficial (Herman and Clark, 2023; Kenny, 2006), PT might help musicians learn to perform well-despite experiencing anxiety. Therefore, it is certainly interesting to explore whether PT could also be valuable in a music setting and to investigate the methods that can effectively induce pressure in this context.

First, it is important to establish whether and to what degree PT is already part and parcel of practice of musicians. Mornell et al. (2020) investigated practice strategies of elite musicians and found an emphasis on quantity of practice with a focus on technical aspects of playing, repetition and making hours, rather than quality of practice focused on preparation for performances. Similar results were found by Kegelaers et al. (2022) who investigated practice and performance strategies of emerging professional musicians. Although, large variations were found among participants, they generally used long-term generic practice goals, rather than very specific SMART goals for individual practice sessions in preparation of a mock audition that was part of their program. Participants also indicated “to focus primarily on technical or physical aspects of playing (e.g., fingerings) to control their attention,” while such an internal focus of attention “might actually be detrimental for performance quality under pressure as it tends to disrupt automated movement patterns” (Mornell and Wulf, 2019; Kegelaers et al., 2022, p. 186). Furthermore, participants reported to have several practice strategies to deal with high pressure but they showed great variation in the use of such strategies and had a preference for long practice hours. As such, these elite musicians did not structurally incorporate PT in their practice strategies.

When training under pressure, it is advised to create a training situation that resembles the high-pressure situation as much as possible (Kegelaers and Oudejans, 2024). However, this does not imply that the anxiety experienced during practice needs to match the intensity of real-life situations. Practicing under mild levels of pressure can still enhance performance in more demanding situations with higher levels of anxiety (Cabral et al., 2024; Oudejans and Pijpers, 2010). One of the most stressful, yet inevitable performance situations in a musician's career is undoubtedly the audition (Kenny et al., 2014). Therefore, preparing for an audition may be most effective when it involves mild levels of pressure like performing in front of a small audience (e.g., family, friends, and colleagues). Kegelaers et al. (2022) investigated what practice and performance management strategies experienced musicians use in order to prepare themselves for an orchestra audition. They showed that their participants used try-outs to simulate the anxiety of performing in front of others, thereby developing the skills and confidence necessary to excel in a real-life audition. Furthermore, research showed that music students expressed their wish for more opportunities for try-outs, so they could improve their performance skills and to learn to deal with performance anxiety (Fehm and Schmidt, 2006). Moreover, Candia et al. (2023) investigated the impact of repeated stage exposure on performance quality, as well as on heart-rate and subjective measures of MPA. In their study, they included 18 string players who experienced MPA and required them to perform a challenging piece of music three times in front of an audience on the same day. The results revealed a significant decrease in heart rate, playing errors, and subjective measures of MPA from the first to the third performance without additional off-stage rehearsals. Based on these findings, the authors highlight the importance of on-stage practice in enhancing live performance quality and preventing the risk of stress-related mental problems. Consequently, it can be hypothesized that regularly participating in try-outs and mock auditions may enhance the ability to perform under pressure, while, importantly, also alleviating some of the mental health issues due to MPA mentioned by Kenny (2011) and Kenny et al. (2014). As such, proper implementation of PT would lead to sustainable high performance under pressure, while maintaining mental health and wellbeing.

### 6.1 Simulation training

Although mock auditions and try-outs offer high ecological validity and therefore appear to be particularly suitable for PT, organizing mock auditions or try-outs can be costly, potentially challenging to arrange, and therefore not always accessible in everyday life. One possibility to overcome these challenges is making use of simulation training. Recognizing the lack of training opportunities for musicians, a performance simulator was developed at the Royal College of Music in London. A performance simulator is a virtual environment, including simulations of auditions and recitals, which allows students to rehearse for potentially stressful situations in a safe environment. Williamon et al. (2014) conducted a study to explore the potential use of these simulation environments for enhancing musicians' learning and performance. They included 11 violinists who participated in both the recital and the audition simulation, after which they filled out a questionnaire about their experiences. Additionally, they compared self-reported state anxiety and heart rate variability between the simulations and the corresponding real auditions. Results showed that both simulated environments provided a realistic performance experience and were deemed particularly useful for enhancing performance skills and managing performance anxiety. Moreover, no significant differences were found in state anxiety and heart rate variability between the simulated and real auditions, indicating that similar psychological and physiological stress responses were evoked by the simulator as in real-life.

In a follow-up study, Aufegger et al. (2017) conducted a focus-group interview with students about their experiences practicing with the same performance simulator. These students reported similar feelings of nervousness prior to entering the simulation, as if it were a live performance or audition. Furthermore, they acknowledged that repeated exposure in the performance simulator could help overcome issues like performance anxiety and better prepare individuals both physically and mentally for real performance situations. Finally, the students suggested that the performance simulator could be effectively combined with other performance enhancement interventions to optimize physical and mental responses in performance situations. This aligns with a recent literature review by Kegelaers and Oudejans (2024) and a meta-analysis of Low et al. (2021), who indicated that PT can be particularly effective when combined with other mental techniques such as goal-setting, imagery, self-talk, and mindfulness, allowing these skills to be practiced and applied under pressure in an ecologically valid setting. Creating opportunities to apply and train these mental skills in actual performance settings can be challenging due to the costs and limited accessibility of real performance situations. However, simulation training provides a valuable alternative, enabling these skills to be developed and applied in a situation where they are most needed (Aufegger et al., 2017).

### 6.2 Virtual reality

Another promising approach to train under pressure may involve using Virtual Reality (VR), a method that allows users to interact with and immerse themselves in computer-generated environments in a naturalistic manner, providing opportunities to expose themselves to anxiety-inducing situations (Riva et al., 2016). As such, like the performance simulator, VR could serve as a bridge between imaginary and *in-vivo* exposure, functioning as a valuable tool for creating pressure to develop performance skills, as it has already proven effective in other high-pressure domains (Neumann et al., 2018; Pallavicini et al., 2016; Renganayagalu et al., 2021). In these fields, VR has been shown to improve responses to unexpected events, enhance psychological resilience, and boost mental performance under pressure (for a meta-analysis, see Richlan et al., 2023).

In order to investigate the suitability of VR in the field of music, Osborne et al. (2022) conducted a case study in which they investigated whether virtual reality can be used to evoke the same physiological and subjective fight-or-flight response that musicians often experience when performing in high-stress environments. They included a highly trained violinist with over 30 years of professional experience and guided him remotely through the VR routine. The results showed that this VR routine can create the situational stress needed to elicit similar physical and psychological responses as when performing under pressure. The authors argued that because of this, VR gives musicians a safe and realistic environment to train under pressure and practice psychological skills for reducing MPA. This aligns with a previous case study that examined whether a virtual musical performance environment could elicit physiological reactions of heightened anxiety in three undergraduate saxophonists. The results confirmed that a virtual setting is successful in provoking anxiety, making it a promising tool for MPA interventions (Orman, 2004). Additionally, no access to a performance lab is needed, which makes practicing under pressure with the use of VR more easily accessible to musicians.

Despite its potential, only a few studies have investigated the use and effectiveness of VR in enhancing musicians' performance. To illustrate, Bissonnette et al. (2015) examined the impact of virtual reality exposure training on music students experiencing performance anxiety. Participants were randomly assigned to either a control group (*n* = 8) or a virtual training group (*n* = 9) and were required to perform in two separate recitals 3 weeks apart. Between both recitals, the virtual training group took part in six 1-h sessions of virtual reality exposure training. The results indicated a reduction in performance anxiety among students with high levels of trait anxiety, and a significant improvement in performance quality in the experimental group, compared to the control group. In a follow-up study, Bissonnette et al. (2016) asked participants to comment on their experiences during the virtual reality exposure training sessions. Participants remarked that the virtual environment used during training closely resembled a real concert setting. They appreciated this resemblance, stating that it helped them prepare for actual performances by giving them the sensation of having already played in front of an audience and aiding them in identifying strategies to manage performance pressure.

### 6.3 Pressure training integrated in performance psychology interventions

Over the last years, performance psychology interventions have increasingly been studied in the field of music, mainly focusing on developing strategies to cope with the pressure. For instance, Mornell and Wulf (2019) showed that adopting an external focus of attention while performing in front of an audience (i.e., with increased pressure) resulted in superior musical expression and technical precision compared to an internal focus or control conditions. Furthermore, promising effects of mental skills training were observed on self-efficacy (Lubert and Gröpel, 2022), perceived MPA (Hoffman and Hanrahan, 2012), focus and present moment awareness (Osborne et al., 2014), and performance quality (Cohen and Bodner, 2019). However, in music psychology research, only a limited number of studies have combined performance psychology interventions with PT, even though this combination has been proven effective in other high-pressure domains for enhancing performance, increasing awareness, and developing mental performance skills (Kegelaers and Oudejans, 2024; Low et al., 2021).

To explore whether an intervention based on principles from the fields of sports and police work, including PT, could also benefit musicians, Bakker et al. (2024) conducted the Study Lab (SL) research project at the Conservatory of Amsterdam (see also Bakker et al., 2016). In 2015 and 2016, a total of 12 music students participated in this project, in which they had to prepare for a challenging recital in 10–14 days. At the start of the SL, the students received an introduction, music for their recital, several lectures and a booklet with assignments about deliberate practice methods, external focus of attention, and imagery. In addition, during the SL, two try-outs were held where the students could perform parts of their recital for each other, allowing them to practice their performance under pressure. At the end of the SL, the students performed in front of a live audience, and their teachers who also assessed the quality of the performance. Furthermore, the students filled out evaluation questionnaires and participated in an exit interview. Results showed that the teachers assessed the musicians' overall performances as better than expected. Furthermore, the students reported that they learned a lot during the SL, and they rated the two try-outs as valuable opportunities to train under pressure.

While try-outs, a performance lab or using VR may be a valuable addition to a musician's practice routine, it is not always readily accessible to everyone. Considering that musicians often rehearse alone for multiple hours each day (Jørgensen, 2008), it raises the question of what methods musicians can use themselves in their own practice sessions to improve their performances under pressure. Kegelaers and Oudejans (2020) tried to address this gap by conducting a study including transitional elite and elite musicians to evaluate the process of a performance psychology intervention. They used a performance psychology intervention that was previously successfully used with music students (Bakker et al., 2016). The aim of this intervention was to improve musicians' quality of practice and performance preparation by using performance psychology principles. Eight transitioning elite and seven elite musicians were included, who all followed one educational session and four workshops, covering principles of deliberate practice, imagery, focus of attention, and performing under pressure. After the workshops, weekly individual monitoring sessions were held for 10 weeks by the research assistants. The intervention ended with a mock audition for the transitioning elite musicians and a week of orchestra performances for the elite musicians, followed by concluding individual interviews. Results showed that this intervention led to a re-examination of practice habits. It increased awareness, practice efficiency and focus, and resulted in structured and goal-directed practice routines, enhanced attention for physical aspects of playing, and a more proactive approach to performances. Especially the transitioning elite musicians stated that the workshops helped them to be more conscious in preparing for stressful performances, mainly by using imagery, scenario planning, and planned disruptions. Furthermore, they highlighted the importance of the monitoring sessions, which allowed them to ask questions and discuss challenges in applying the methods and kept them motivated to continue using the learned principles. Overall, this study provides a comprehensive overview of applying a performance psychology intervention in a music context, including workshops, methods for pressure training, and incorporating regularly scheduled reflection sessions. This aligns with developments in sports psychology, which advocate that, when implementing PT, one should not only practice under pressure but also provide tools to handle that pressure and use moments of reflection to enhance the learning experience (Fletcher and Arnold, 2021; Kegelaers et al., 2021; Low et al., 2021). Again, note that next to performance enhancement under pressure, proper and successful implementation of the methods would also help in maintaining mental health and wellbeing, by alleviating the negative effects of MPA. Thus, PT does not only allow for performance enhancements under stress but also for musicians to maintain their wellbeing.

## 7 Future directions and recommendations

This narrative review aimed to explore the potential of PT in music settings to help musicians deal with MPA and learn to perform better under pressure, despite MPA. Research indicates that the underlying mechanisms for choking under pressure are similar between musicians and performers in other high-pressure domains. This suggests that PT, an intervention that is proven to be effective in sports and police work (Low et al., 2021), might also have a positive effect on musicians who struggle to perform at a high level under pressure. Over the years, there has been increasing interest in exploring methods to enhance performance under pressure in music contexts, leading to the development of several PT approaches. Examples include the development of a simulation lab for practicing under pressure, studies investigating the use of VR, organizing try-outs and mock auditions, and implementing interventions incorporating methods to induce pressure during private practice sessions. Although the number of studies investigating the effectiveness of these approaches on performance quality are limited, leaving multiple aspects for future exploration, studies focusing on musicians' experiences have demonstrated that PT can be especially beneficial for enhancing performance skills, preparing for performances, and managing performance anxiety. To reiterate a key point, PT should never be used as an excuse to expose musicians (or any other performer) to any environmental hazard or transgressive behavior (see Kegelaers et al., 2020; Kegelaers and Oudejans, 2024). The specific kind of pressure, its according intensity, and the desired outcome should be clearly defined and ideally be established empirically before conducting PT (see Hill et al., 2024), and both introduced and framed and evaluated (debriefed) properly with the performers themselves. Doing the latter is in line with the suggestion by Herman and Clark (2023) to not just focus on the relationship between MPA and performance but also on differences in interpretation and what MPA means for the performer (i.e., the experiences of the performer with pressure and MPA).

Future research should focus on expanding the understanding of the underlying mechanisms of choking in musicians. Research in the field of sports and law enforcement investigated choking by observing behavioral and cognitive characteristics such as gaze behavior, shooting accuracy, and thoughts (Nieuwenhuys and Oudejans, 2011; Wilson et al., 2009). However, research on musicians only focused on cognitive characteristics. It would be fruitful to gain a better overview of what factors cause distractions for musicians when performing under pressure. For example, gaze behavior when reading sheet music or standing in front of a large audience could indicate how attentional shifts express themselves. Then, it might be possible to develop more specific and accurate targeted interventions for improving attentional processes while performing under pressure. Furthermore, only a limited number of studies investigated the effectiveness of PT alone or in combination with other interventions on the quality of music performance. It must be acknowledged that objectively measuring the quality of a musical performance is more difficult than, for example, assessing shooting accuracy in police work, particularly since evaluations are often influenced by subjectivity and rater intuition rather than systematic procedures (Wesolowski et al., 2015). However, to determine the effectiveness of PT in music on preventing choking and enhancing performance, its influence on performance quality must be investigated. This also includes identifying the optimal frequency (i.e., how often) and timing (e.g., just before an audition or regularly) of PT. Finally, in sport psychology research, it has been shown that coaches can play an important role in administering PT through the use of planned disruptions (Kegelaers et al., 2020). It would be interesting to develop planned disruption more specific for (student) musicians and music settings, and to investigate the role of teachers and conductors in this process. In this way, PT becomes more accessible and can be integrated more easily into the daily practice of (student) musicians.

Based on the reviewed literature, several recommendations can be offered for musicians seeking to enhance their ability to perform under pressure as well as for teachers and institutions working with these musicians.

For musicians:

- It is recommended that musicians deliberately arrange try-outs for themselves to practice their music and mental performance skills while under pressure.
- Musicians can use techniques as planned disruptions, imagery, and scenario planning, for a more conscious way of preparing themselves for a performance.
- After the performance, moments of reflection can be employed to determine what went well and identify areas for further improvement.
- Musicians are advised to seek help if they experience difficulty applying these principles by themselves. Working with a sport and performance psychologist can help musicians develop techniques to improve their mental skills, practice these techniques under pressure, and reflect on their experiences afterwards.

For teachers:

- Research shows that there is still much controversy at conservatories around mental health issues, such as MPA, and students often perceive a lack of support to deal with these (Perkins et al., 2017). In addition, studies indicate that musicians value the opportunity to share their experiences and concerns related to the mental aspects of performing (Kegelaers and Oudejans, 2020). Therefore, especially for teachers, it is important to discuss performing under pressure with their students to normalize the existence of performance anxiety, and to emphasize that performing under pressure is a skill that can be actively developed and improved with practice.
- Music teachers should reduce the stigma surrounding MPA through open dialogues and creating supportive environments. Furthermore, they should refer students to a professional sport and performance psychologist if they notice that the students' struggles with performing under pressure exceed their own expertise. This allows the student to work with a psychologist to improve and develop their mental performance skills.

For music institutions:

- Music institutions should recognize the importance of providing musicians with opportunities to practice under pressure and facilitate try-outs where possible (cf. Bakker et al., 2024; Candia et al., 2023).
- Music institutions, such as conservatories, could invest in the development of a performance lab or provide access to VR equipment, enabling musicians to use these resources as part of their preparation for important performances. Next to the Study lab project (Bakker et al., 2016, 2024) examples of how to include specific programs into curricula of conservatories are described in Hildebrandt (2009). These examples are not specifically focused at pressure training but more generally at the processes and mechanisms of motor learning and stage disposition in music.
- To ensure that PT in a performance lab or with VR is implemented in a way that allows musicians to benefit the most, music institutions are advised to work with sport and performance psychologists. These professionals can work with musicians to enhance their mental performance skills and develop self-awareness through reflection on their practice sessions (Fletcher and Arnold, 2021).

## 8 Conclusion

In conclusion, PT holds considerable promise for enhancing performance skills in music settings and improving performance preparation. It is crucial to recognize the potential challenges in developing practical and effective PT methods in music settings, particularly the need to balance pressure training with maintaining a safe learning environment. When applied deliberately, safely, and ethically, PT can serve as a valuable addition to existing interventions. It provides musicians with the opportunity to improve and develop their performance skills under pressure, despite their anxiety. Consequently, pressure training can elevate performance quality and support musicians in reaching higher levels of musical excellence on stage while preserving joy, motivation and, in general, mental health, and wellbeing.
