# Supplementary material for: Facing the fear: a narrative review on the potential of pressure training in music
Source: Front Psychol. 2024 Dec 13;15:1501014. doi: 10.3389/fpsyg.2024.1501014 (PMC11671778; doi:10.3389/fpsyg.2024.1501014)
Supplement: Supplementary file 1 [file Table_1.docx]

**Appendix: Search strategy for narrative review on pressure training in music**

1. Search for information regarding the definition, prevalence and treatment of Music Performance Anxiety.

Keywords:

*Music performance anxiety AND definition OR prevalence OR treatment.*

The authors D.T. Kenny and M.S. Osborne were also included in this search, due to their expertise on music performance anxiety.

1. Search for information on underlying mechanisms of choking under pressure.

Keywords:

*Choking under pressure AND sports OR music OR police work*

*Attentional control theory AND sports OR police work*

*The Integrated Model of Anxiety and Perceptual-Motor Performance AND choking under pressure*

The authors R.R.D. Oudejans and A. Nieuwenhuys were also included in this search, because of their expertise on this topic. This search provided multiple studies on the underlying mechanisms of choking under pressure in the domain of sports, police work, and music, five of which were discussed in the review.

1. Search for studies on pressure training in sports and police work

Since there are few studies on the use of pressure training in music, the focus was first on its application in other performance domains, such as sports and police work. Based on R.R.D. Oudejans' expertise in research on the implementation of pressure training in sports and policing, the following authors were included in the search: D. Fletcher and R. Arnold, S. Kent, W.R. Low, J. Kegelaers, and R.R.D. Oudejans.

Keywords:

*Pressure training AND sports OR police work*

*Pressure training AND sports AND planned disruptions OR implementation*

After the initial search on this subject, the following meta-analysis and narrative review were used to identify more and relevant references on the subject of pressure training in sports and other domains:

*Low, W. R., Sandercock, G. R. H., Freeman, P., Winter, M. E., Butt, J., & Maynard, I. (2021). Pressure training for performance domains: A meta-analysis. Sport, Exercise, and Performance Psychology, 10(1), 149.* doi: 10.1037/spy0000202

|  |
| --- |

*Kegelaers, J., & Oudejans, R. R.D. (2024). Pressure makes diamonds? A narrative review on the application of pressure training in high-performance sports. International Journal of Sport and Exercise Psychology, 22(1), 141-159.* doi: 10.1080/1612197X.2022.2134436

1. Search for studies on pressure training in music

First, a general search was conducted on interventions for music performance anxiety to gain an overview of recent developments in this area. The author A. Williamon was included in the search due to his involvement in the development of the simulation lab at the Royal College of Music in London. Additionally, the search covered various forms of pressure training combined with MPA, such as virtual reality and mock auditions. Based on the expertise of R.R.D. Oudejans, two studies involving a combination of performance interventions and try-outs (practice under pressure) were also included.

Keywords:

*Music AND mental skills training OR performance training*

*Music performance anxiety AND virtual reality OR mock auditions OR performance training*

*Music performance anxiety AND performance simulation AND Williamon*

*Music performance anxiety AND planned disruptions AND Kegelaers & Oudejans*
